# Dr. W. H. Dwinelle

**Published:** 1885-03

**Authors:** 


### DR. W. H. DWINELLE.

At the dinner of the Odontological Society, on the evening of the 18th ult., a painful announcement was made. Dr. Dwinelle, it was said, had that day been suddenly taken alarmingly ill, and the gravest consequences were feared. There were very many anxious enquiries made concerning his condition during the day or two following, and many of the guests of the society departed without obtaining any assurance that the worst was over. It gives us great pleasure to assure them, on the authority of a telegram from Dr. Kingsley, that he has so far recovered that no immediate apprehensions need be felt. Dr. Dwinelle, like the lamented McQuillen and Webb, has always worked harder for his professional brethren than for himself, and some exhaustive labors and anxieties lately undertaken on their behalf, added to his professional work, were too much for his strength, and resulted in a temporary breaking down of his nervous system. Quiet and rest (if he can be prevailed upon to take them) will, it is hoped, result in his entire restoration to health.

